# Age at Autism Spectrum Disorder (ASD) Diagnosis by Race, Ethnicity, and Primary Household Language Among Children with Special Health Care Needs, United States, 2009–2010

**DOI:** 10.1007/s10995-015-1683-4

**Published:** 2015-02-21

**Authors:** Heejoo Jo, Laura A. Schieve, Catherine E. Rice, Marshalyn Yeargin-Allsopp, Lin H. Tian, Stephen J. Blumberg, Michael D. Kogan, Coleen A. Boyle

**Affiliations:** 1 Division of Birth Defects and Developmental Disabilities, National Center on Birth Defects and Developmental Disabilities, Centers for Disease Control and Prevention, MS-E86, 1600 Clifton Road, Atlanta, GA 30333 USA; 2 National Center for Health Statistics, Centers for Disease Control and Prevention, Hyattsville, MD 20782 USA; 3 Maternal and Child Health Bureau, Health Resources and Services Administration, Rockville, MD 20857 USA

**Keywords:** Autism, Age at diagnosis, Race/ethnicity, Prevalence

## Abstract

We examined prevalence of diagnosed autism spectrum disorder (ASD) and age at diagnosis according to child’s race/ethnicity and primary household language. From the 2009–2010 National Survey of Children with Special Health Care Needs, we identified 2729 3–17-year-old US children whose parent reported a current ASD diagnosis. We compared ASD prevalence, mean diagnosis age, and percentage with later diagnoses (≥5 years) across racial/ethnic/primary household language groups: non-Hispanic-white, any language (NHW); non-Hispanic-black, any language (NHB); Hispanic-any-race, English (Hispanic-English); and Hispanic-any-race, other language (Hispanic-Other). We assessed findings by parent-reported ASD severity level and adjusted for family sociodemographics. ASD prevalence estimates were 15.3 (NHW), 10.4 (NHB), 14.1 (Hispanic-English), and 5.2 (Hispanic-Other) per 1000 children. Mean diagnosis age was comparable across racial/ethnic/language groups for 3–4-year-olds. For 5–17-year-olds, diagnosis age varied by race/ethnicity/language and also by ASD severity. In this group, NHW children with mild/moderate ASD had a significantly higher proportion (50.8 %) of later diagnoses than NHB (33.5 %) or Hispanic-Other children (18.0 %). However, NHW children with severe ASD had a comparable or lower (albeit non-significant) proportion (16.4 %) of later diagnoses than NHB (37.8 %), Hispanic-English (30.8 %), and Hispanic-Other children (12.0 %). While NHW children have comparable ASD prevalence and diagnosis age distributions as Hispanic-English children, they have both higher prevalence and proportion of later diagnoses than NHB and Hispanic-Other children. The diagnosis age findings were limited to mild/moderate cases only. Thus, the prevalence disparity might be primarily driven by under-representation (potentially under-identification) of older children with mild/moderate ASD in the two minority groups.

## Introduction

Autism spectrum disorder (ASD) is characterized by impairments in social interactions and communication and restricted, repetitive, and stereotyped patterns of behavior [1]. ASD symptoms are often noted early in life and diagnosis can occur as early as 2 years of age [1, 2]. Previous studies suggest early diagnosis is important because early interventions may be more effective [3–6]. Recent studies suggest that ASD prevalence has increased [7–16] and ASD diagnosis age has decreased in US children [7, 9, 17–20]; however, racial/ethnic differences remain [7–9, 11, 12, 15, 17–29].

The most recent US population-based ASD estimates from both the 2010 Autism and Developmental Disabilities Monitoring (ADDM) Network (1.5 %) and the 2011–2012 National Survey of Children’s Health (NSCH) (2 %) represented substantial increases from prevalence estimates just a few years earlier [7, 9]. Additionally, both ADDM and NSCH studies have shown that non-Hispanic-white children had higher ASD prevalence than non-Hispanic-black and Hispanic children [8, 11], and an NSCH study documented that Hispanic children with foreign-born parents had a substantially lower prevalence than Hispanic children with US-born parents and all non-Hispanic-white children [26].

Several studies suggest that ASD diagnosis age has been decreasing, overall, among US children. While in the 2002 ADDM, the median age at first diagnosis was 5.7 years, the mean age in 2006 was 4.4 years [15, 17]. Likewise, the mean age of receipt of ASD services from the California Department of Developmental Services decreased from 6.9 to 3.3 years from 1987 to 1994 [18]. Previous studies also reported that, as expected, diagnosis age varies by ASD subtype, with notably higher diagnosis ages for Asperger’s disorder than autistic disorder [7, 20], and that later ASD diagnosis age is associated with non-Hispanic-black and Hispanic race/ethnicity [24, 29], low income [20, 24], lack of awareness among healthcare professionals and parents [21, 23], certain clinical presentations [12], and physician behaviors in screening practices [19, 20, 22, 27, 28].

Beyond general studies of predictive factors, there have been few in-depth population-based studies of ASD diagnosis age by child’s race/ethnicity. Past studies were limited by small sample size and have not accounted for variation within the Hispanic subgroup. In this study, we compare ASD diagnosis age across racial/ethnic groups in a population-based sample from the 2009–2010 National Survey of Children with Special Health Care Needs (NS-CSHCN). Additionally, we study Hispanic children within subgroups based on primary household language, which was previously found to be closely correlated with parents’ birthplace and thus, an indicator of the autism prevalence heterogeneity among Hispanic children [26]. Within all racial/ethnic groups, we also consider child’s ASD severity as a potential predictor or modifier of diagnosis age, and assess the variation of ASD prevalence estimates by both race/ethnicity and diagnosis age.

## Methods

### Study Sample

The 2009–2010 NS-CSHCN was a random-digit-dial telephone survey conducted by the National Center for Health Statistics. Households were the primary sampling units. In each selected household with children, a parent/guardian was administered a screening instrument to determine if any children had special healthcare needs. Special healthcare needs were defined as meeting one or more criteria in association with a long-term health condition: ongoing need for prescription medications; need for more medical care, mental health, or educational services than usual for most same-aged children; limited abilities in comparison to most same-aged children; need for special therapy, such as physical, occupational, or speech therapy; and/or having an emotional, developmental, or behavioral problem necessitating treatment or counseling [30]. If one child was identified as having special healthcare needs, s/he was selected to be the subject of a detailed parent/guardian interview. If more than one child was identified, one was randomly selected to be the subject of the interview.

The final NS-CSHCN data files include screening data on 371,617 children. Of these, 40,242 CSHCN were selected and detailed interviews were completed. The overall response rate was 25.5 %. Most nonresponse occurred due to no answer or person answering did not identify whether a child lived in the household; this nonresponse was more common for cell-phones than for landlines. Among households known to include a child with special healthcare needs, the interview completion rate was 80.8 % [31].

Of the 40,242 CSHCN with completed interviews, 38,034 were aged 3–17 years. For this analysis, we initially selected all 3025 CSHCN aged 3–17 years with parent-report of ASD. We did not include children aged <3 years because many children with ASDs are not diagnosed earlier [7, 15, 17, 20]. We excluded 296 Asian, Native Hawaiian/Pacific Islander, and multiple-race children because small sample sizes in these subgroups precluded race-specific analyses. Our final sample included 2729 non-Hispanic-white, non-Hispanic-black, and Hispanic children with ASD.

### ASD Ascertainment

ASD was ascertained using two questions: “Has a doctor or other health care provider ever told you that [CHILD] had autism, Asperger’s disorder, pervasive developmental disorder, or other autism spectrum disorder?” and if YES, “Does [CHILD] currently have autism or an autism spectrum disorder?” Children whose parents answered affirmatively to *both* questions were classified as having ASD. Because 23 % of children reported to have a past ASD diagnosis were not reported to have ASD currently, this ASD definition might be conservative.

### Ascertainment of ASD Diagnosis Age and Severity

Parents of children classified as having “current” ASD were further asked to describe their child’s autism or ASD as either mild, moderate, or severe and were asked their child’s age at first autism/ASD diagnosis. No further instructions were given in regards to assigning the severity level. We initially analyzed diagnosis age as a continuous variable and an ordinal variable: 3–4, 5–8, 9–12 and ≥13 years. Because of sample size constraints, detailed analyses related to profile and predictors of diagnosis age used a dichotomized outcome measure: later diagnosis age (≥5 years, Yes/No). This cut-point was chosen based on sample size and recent US data indicating mean ASD diagnosis age was 4.4 years [17].

Parents’ perceptions of ASD severity were dichotomized as severe versus mild/moderate. Mild and moderate responses were combined because an empiric assessment indicated they were similarly associated with diagnosis age. We also assessed a second indicator of severity, parent-report of intellectual disability (ID) diagnosis (analogous question series as ASD).

### Race/Ethnicity/Language and Other Variables of Interest

Our outcome of interest was diagnosis age and the main risk/predictive factor of interest was race/ethnicity. Four racial/ethnic and primary household language groups were created: non-Hispanic-white, any language (NHW); non-Hispanic-black, any language (NHB); Hispanic-any-race, English (Hispanic-English); and Hispanic-any-race, other language (Hispanic-Other).

Other demographic characteristics included child’s age at survey, child’s sex, highest education of parent/guardian, household income relative to the federal poverty level, total number of children in household, type of health insurance, and family structure. Additionally, we assessed several co-occurring health conditions previously reported to be more common in children with ASDs: difficulty with repeated chronic physical pain (including headaches), asthma, epilepsy/other seizure disorder, allergies (any type), food allergies, and Attention-Deficit/Hyperactivity Disorder (ADD/ADHD) [32]. We did not examine several other co-occurring conditions (e.g. diabetes) due to sample size constraints.

### Statistical Analyses

Within each race/ethnicity/language group, ASD prevalence was estimated as the number of CSHCN who currently had ASD, divided by the total number of 3–17-year-old children who were included in the initial household screening sample. Thus, the denominator included both CSHCN and children without special healthcare needs. Because children first classified as having special healthcare needs were the subjects of the detailed interviews, information on a child’s ASD diagnosis was only ascertained on these selected children. Although prevalence estimates were weighted to be representative of the US population of non-institutionalized children aged 3–17 years, some ASD cases might have been missed if a parent did not first indicate a special healthcare need. However, independent assessments of data from other nationally representative surveys indicate that 94 % of children whose parents respond affirmatively to the ASD questions are classified as CSHCN [33]. In addition to race/ethnicity/language group, we calculated ASD prevalence within strata based on timing (age) of ASD diagnosis and severity.

We assessed mean and percentage distribution of ASD diagnosis age within strata defined based on both race/ethnicity/language and child’s age at survey. The latter stratification was needed to control for birth-cohort-related factors or differential time frames in which a child could have received a diagnosis.

The remainder of analyses were limited to children aged 5–17 years because younger children, by definition, did not have a later ASD diagnosis. We assessed ASD severity and sociodemographic factors as potential predictors of later diagnoses, and the profile of children with earlier versus later diagnoses in terms of their co-occurring health conditions.

In various analyses, we used *t* tests and Chi square tests to compare group differences in means and percentage distributions, respectively. We calculated unadjusted odds ratios (ORs) with 95 % confidence intervals (CIs) in which the odds of later diagnoses within each race/ethnicity/language group were compared to those for NHWs. We similarly calculated stratum-specific ORs. We tested for significant interactions between race/ethnicity/language and each severity indicator using likelihood ratio tests.

Because of sample size constraints in some subgroups, we calculated relative standard errors (RSE, standard error/prevalence estimate ×100) for each estimate and indicate those that have a moderately high variability (30–50 %) or high variability (>50–60 %). We do not present estimates where RSE is >60 %.

Adjusted odds ratios (aORs) for associations between later ASD diagnoses and sociodemographic variables were computed using multivariable logistic regression. We constructed two models: the first model included race/ethnicity/language and the aforementioned other sociodemographic factors as independent variables; the second model additionally included ASD severity and an interaction term to examine the potential effect modification between race/ethnicity/language and ASD severity level. We additionally evaluated the first model stratified on both ASD severity and co-occurring ID.

All estimates were weighted to obtain nationally representative estimates [31]. We used multiply-imputed data for child’s race/ethnicity, primary household language, highest education of parents/guardians, and household income [33]. Analyses were conducted using SAS-callable SUDAAN (Research Triangle Institute, Research Triangle Park, NC) to account for the complex sampling design.

Human subjects review was not required for this study since this was a secondary analysis of de-identified datasets.

## Results

### Characteristics of CSHCN with ASD

**Fig. 1:**
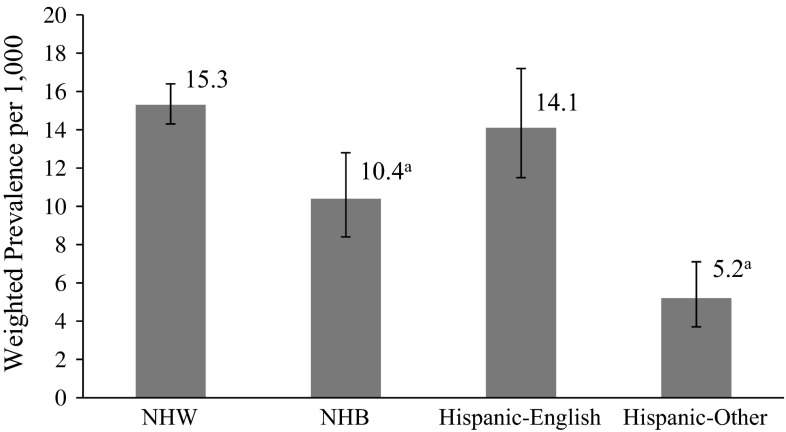
Estimated prevalence of CSHCN aged 3–17 years with ASD by race/ethnicity/language, United States, 2009–2010. Racial/ethnic and primary household language groups were categorized as: non-Hispanic-white, any language (NHW); non-Hispanic-black, any language (NHB); Hispanic-any race, English (Hispanic-English); and Hispanic-any race, other language (Hispanic-Other). ^a^Prevalence estimate is significantly different from NHW children

In this nationally representative study, the estimated prevalence of CSHCN with diagnosed ASD was 15.3 per 1000 NHW children aged 3–17 years (Fig. 1). In comparison, Hispanic-English children had a similar estimate, while NHB and Hispanic-Other children had significantly lower ASD estimates.

Among CSHCN with ASD, distributions of demographic characteristics varied across race/ethnicity/language groups (Table 1). Age at survey was markedly younger and household education and income levels were notably lower for Hispanic-Other children compared to other groups. Hispanic-Other children had the highest proportion of no health insurance and NHB children had the highest proportion of public-only health insurance. While most children lived in two-parent biological/adopted households, 40 % of NHB children lived in single-mother-households, twice that of all other groups. Neither child sex nor number of children in the household varied significantly across race/ethnicity/language groups; nor did either of the ASD severity indicators (although non-significant variation was noted).

**Table 1:** Percentage distribution^a^ of selected demographic characteristics of CSHCN aged 3–17 years with ASD by race/ethnicity/language, United States, 2009–2010

	Total (n = 2729) (%)	NHW (n = 2192) (%)	NHB (n = 208) (%)	Hispanic-English (n = 256) (%)	Hispanic-Other (n = 73) (%)	*P* value^b^
Age (years)						
3–5	17.2	13.4	23.7	21.5	45.5	<0.005
6–10	38.7	38.1	39.7	46.1	24.7	
11–17	44.1	48.5	36.6	32.4	29.9	
Sex						
Male	80.3	79.8	81.7	81.0	83.5	0.91
Female	19.7	20.2	18.4	19.0	16.5^c^	
Highest education household member						
<High school	8.3	5.4	10.9	15.3	27.1	<0.005
High school	16.0	14.1	19.8	17.1	31.5	
>High school	75.7	80.5	69.3	67.6	41.4	
Household income						
≤200 % Federal poverty level	40.4	34.9	57.6	42.7	73.0	<0.005
>200 % Federal poverty level	59.6	65.1	42.5	57.4	27.0^c^	
Total number of children in household						
1	25.3	24.8	28.9	22.5	31.1	0.40
2	41.5	42.6	42.3	40.3	27.1	
3+	33.2	32.6	28.8	37.3	41.8	
Health insurance						
Private/both/other	66.1	72.3	41.8	61.1	47.1	<0.005
Public	31.2	25.9	52.5	35.7	45.0	
Uninsured	2.7	1.8	5.7^c^	3.2^d^	7.9^d^	
Family structure						
Two-parent biological/adopted	63.0	67.0	44.1	56.3	68.9	<0.005
Two-parent stepfamily	8.4	7.8	8.6	13.0	–^e^	
Single mother, no father present	20.4	16.7	40.2	22.0	20.8	
Other	8.3	8.5	7.1^c^	8.7^b^	6.9^d^	
ASD severity						
Mild	49.9	50.3	44.4	51.5	53.3	0.23
Moderate	35.9	36.0	34.5	40.4	25.9	
Severe	14.2	13.7	21.1	8.1^c^	20.7^c^	
Intellectual disability						
Yes	26.5	23.8	34.0	31.8	33.6	0.10
No	73.5	76.3	66.0	68.2	66.4	

^a^Weighted percentages

^b^Chi square test of independence between each characteristic and race/ethnicity/language

^c^Relative standard error (RSE) is 30–50 %

^d^RSE is >50–60 % indicating the variance is large and thus estimate should be interpreted cautiously

^e^Estimate not presented if RSE was >60 %

### Distribution of Diagnosis Age by Race/Ethnicity/Language

Within each race/ethnicity/language group except Hispanic-Other, diagnosis age increased with each successive increase in child’s age at survey (Table 2). Among 3–4-year-olds, mean ASD diagnosis age was comparable across race/ethnicity/language groups (range 2.3–2.5). Among 5–8-year-olds, there was only slight variation in diagnosis age, although mean age for Hispanic-English children (2.9) was significantly lower than that for NHWs (3.5). Among children aged 9–12 and ≥13 years, mean diagnosis age was comparable for NHW, NHB, and Hispanic-English children (overall means 5.0 and 6.3 for 9–12 and ≥13 years, respectively), while Hispanic-Other children had significantly lower mean diagnosis ages (2.9 and 3.7, respectively).

**Table 2:** Mean age at first diagnosis within each race/ethnicity/language and age at survey group of children aged 3–17 years with ASD, United States, 2009–2010

	Age at survey (years)				
	3–4	5–8	9–12	≥13	5–17
Mean age at first diagnosis (years)					
Overall	2.4	3.4	5.0	6.3	4.9
NHW (n = 2182)	2.3	3.5	5.0	6.5	5.1
NHB (n = 205)	2.4	3.5	5.1	5.8	4.6
Hispanic-English (n = 251)	2.3	2.9^a^	5.3	6.8	4.7
Hispanic-Other (n = 71)	2.5	3.3	2.9^b^	3.7^b^	3.3^b^

^a^ *t* test comparing mean diagnosis age in each race/ethnicity/language group within each age at survey group to NHW children; significant difference between Hispanic-English and NHW children (*P* < 0.05)

^b^ *t* test comparing mean diagnosis age in each race/ethnicity/language group within each age at survey group to NHW children; significant difference between Hispanic-Other and NHW children (*P* < 0.05)

Likewise, among children aged 5–8, 9–12, and ≥13 years, the percentage distributions of the ordinal categories of ASD diagnosis age were fairly similar for NHW, NHB, and Hispanic-English children (Table 3). However, 89 % of Hispanic-Other children aged 9–12 years had an earlier diagnosis compared to 50 % of NHW children, and Hispanic-Other children aged ≥13 years showed a trend (albeit non-significant) toward younger diagnosis age than NHW children (*P* = 0.10).

**Table 3:** Percentage distribution^a^ of age at first diagnosis by race/ethnicity/language and age at survey in children aged 3–17 years with ASD, United States, 2009–2010

Age at first diagnosis (years)	NHW (%)	NHB (%)	Hispanic-English (%)	Hispanic-Other (%)
	Age at survey: 5–8 years			
3–4	74.7	74.9	82.4	84.7
5–8	25.3	25.1	17.6^c^	15.3^d^
	Age at survey: 9–12 years			
3–4	49.9	55.6	48.3	88.9^b^
5–8	38.1	31.1	37.0	–^e^
9–12	12.0	13.3^c^	14.6^c^	0.0^b^
	Age at survey: ≥13 years			
3–4	40.6	57.3	34.5	61.5^c^
5–8	27.9	13.2^c^	37.8	32.3^d^
9–12	23.0	21.9^c^	13.8^d^	–^e^
≥13	8.5	7.5^d^	13.8^d^	0.0
	Age at survey: 5–17 years (n = 2498)			
3–4	54.0	64.7	58.2	79.0^b^
5–8	31.1	23.6	29.9	19.2^b,c^
9–12	12.1	9.7	8.9^c^	–^e^
≥13	2.8	2.0^d^	3.0^c^	0.0^b^

^a^Weighted percentages

^b^Two-tailed *z* test comparing diagnosis age distribution within each race/ethnicity/language group to diagnosis age distribution among NHW; significant difference between Hispanic-Other and NHW children (*P* < 0.05)

^c^Relative standard error (RSE) is 30–50 %

^d^RSE is >50–60 % indicating the variance is large and thus should be interpreted cautiously

^e^Estimate not presented if RSE was >60 %

### Predictors of Later Diagnosis Age, Race/Ethnicity/Language and ASD Severity

Overall, NHW children aged 5–17 years had the highest proportion of later diagnoses while Hispanic-Other had the lowest (Table 4). However, these findings varied by ASD severity. Likelihood ratio tests testing for interactions between race/ethnicity/language and both severity indicators, parent-reported severity and co-occurring ID, were significant. Stratified analyses illustrated the magnitude of these effect modifications. Among children whose parents perceived their severity level as mild/moderate, a higher proportion (50.8 %) of NHW children had later diagnoses than NHB (33.5 %) or Hispanic-Other children (18.0 %). However, among children with severe ASD, a lower (albeit non-significant) proportion of NHW children (16.4 %) had later diagnoses than NHB (37.8 %), and Hispanic-English (30.8 %) children; the proportions appeared fairly comparable for NHW and Hispanic-Other (12.0 %) children. A similar pattern was seen when comparing children with or without co-occurring ID. Overall, the associations between race/ethnicity/language and later diagnoses were not substantially impacted by adjustment for sociodemographic factors. However, the difference in odds of later diagnosis for NHB children with severe ASD increased slightly and was statistically significant.

**Table 4:** Association between race/ethnicity/language and later diagnosis of ASD based on ASD severity indicators among CSHCN aged 5–17 years with ASD, United States, 2009–2010^a^

	Later diagnosis^b^ (%)	Unadjusted OR (95 % CI)	Adjusted OR^c^ (95 % CI)		Later diagnosis^b^ (%)	Unadjusted OR (95 % CI)	Adjusted OR^c^ 95 % CI
Overall							
NHW	46.0	1.00 (Referent)	1.00 (Referent)				
NHB	35.3	0.64 (0.41–1.01)	0.61 (0.39–0.96)				
Hispanic-English	41.8	0.84 (0.54–1.32)	0.77 (0.49–1.19)				
Hispanic-Other	21.0	0.31 (0.14–0.68)	0.33 (0.14–0.76)				
ASD severity: mild/moderate				ASD severity: severe			
NHW	50.8	1.00 (Referent)	1.00 (Referent)	NHW	16.4	1.00 (Referent)	1.00 (Referent)
NHB	33.5	0.49 (0.30–0.79)	0.42 (0.26–0.69)	NHB	37.8	3.10 (0.90–10.71)	3.88 (1.11–13.57)
Hispanic-English	43.5	0.75 (0.47–1.19)	0.63 (0.40–0.99)	Hispanic-English	30.8	2.27 (0.38–13.67)	1.97 (0.39–9.96)
Hispanic-Other	18.0	0.21 (0.09–0.52)	0.20 (0.07–0.53)	Hispanic-Other	12.0	0.70 (0.12–4.19)	1.07 (0.18–6.54)
ASD *without* intellectual disability				ASD *with* intellectual disability			
NHW	49.9	1.00 (Referent)	1.00 (Referent)	NHW	34.0	1.00 (Referent)	1.00 (Referent)
NHB	33.3	0.50 (0.29–0.87)	0.45 (0.26–0.79)	NHB	39.0	1.41 (0.63–3.16)	1.54 (0.68–3.49)
Hispanic-English	40.9	0.69 (0.41–1.17)	0.64 (0.38–1.06)	Hispanic-English	44.1	1.70 (0.70–4.13)	1.33 (0.58–3.05)
Hispanic-Other	26.6	0.36 (0.14–0.94)	0.37 (0.14–1.02)	Hispanic-Other	13.4	0.30 (0.09–1.08)	0.30 (0.07–1.19)

^a^Weighted percentages

^b^Later diagnosis is defined as those diagnosed at age ≥5 years

^c^Model adjusts for education, income, health insurance, family structure, and race/ethnicity/language

### Predictors of Later Diagnosis Age, Sociodemographic Factors

In addition to associations with race/ethnicity/language, the findings from the multivariable models also indicated that children living in two-parent stepfamily households, single-mother-households, or households with other structures were more likely to have a later diagnosis than those living with two biological/adoptive parents (aOR 1.83, 95 % CI 1.35–2.48), and that children living in households with incomes <200 % of the federal poverty level were more likely to have a later diagnosis than those from higher income households (aOR 1.44, 95 % CI 1.02–2.03). Neither household members’ education levels nor child’s type of health insurance were significantly associated with later diagnosis (data not shown).

### Predictors of Later Diagnosis Age, Co-occurring Health Conditions

NHW and NHB children with a later ASD diagnosis were more likely to have co-occurring ADD/ADHD than NHW and NHB children with an earlier diagnosis (Table 5). NHB children with a later diagnosis were also more likely to have difficulty with repeated chronic physical pain. While the reasons varied for this condition, 16 % of NHB children with repeated chronic physical pain also reported as having sickle cell disease.

**Table 5:** Differences in co-occurring health conditions in children aged 5–17 years with ASD based on age at diagnosis and race/ethnicity/language, United States, 2009–2010^a,b^

Age at diagnosis	NHW (%)		NHB (%)		Hispanic-English (%)		Hispanic-Other (%)	
	<5 years	≥5 years	<5 years	≥5 years	<5 years	≥5 years	<5 years	≥5 years
	n = 1000	n = 1030	n = 112	n = 73	n = 135	n = 95	n = 36	n = 17
Difficulty with repeated chronic physical pain, including headaches	28.1	32.3	17.7^c,f^	47.6^f^	33.0	48.0	7.4^c^	8.4^d^
Asthma	20.0	23.8	29.7	35.4	27.1	37.3	14.7^d^	19.6^d^
Epilepsy or other seizure disorder	10.5	8.8	9.7^c^	9.2^d^	10.7^c^	14.1^c^	–^e^	–^e^
Allergies (any type)	48.5	49.0	44.1	64.1	42.7	56.1	27.0^c^	28.7^c^
Food allergies	30.8	24.8	35.3^c^	23.9^c^	51.5	12.2^d^	–^e^	0.0
ADD or ADHD	43.1^g^	71.6^g^	31.8^g^	61.0^g^	41.6	55.3	57.9	74.2

^a^Weighted percentages

^b^These are children with a parent report of ever being told by a doctor or other health care provider that their child had a specific health condition

^c^Relative standard error (RSE) is 30–50 %

^d^RSE is >50–60 % indicating the variance is large and thus should be interpreted cautiously

^e^Estimate not presented if RSE was >60 %

^f^Significant difference in difficulty with repeated chronic physical pain, including headaches between NHB children with earlier diagnosis age (<5 years) and NHB children with later diagnosis age (≥5 years)

^g^Significant difference in ADD or ADHD between NHW children with earlier diagnosis age and NHW children with later diagnosis age; and significant difference in ADD or ADHD between NHB children with earlier diagnosis age and NHB children with later diagnosis age

### Variation in ASD Prevalence by Race/Ethnicity/Language and ASD Severity

**Fig. 2:**
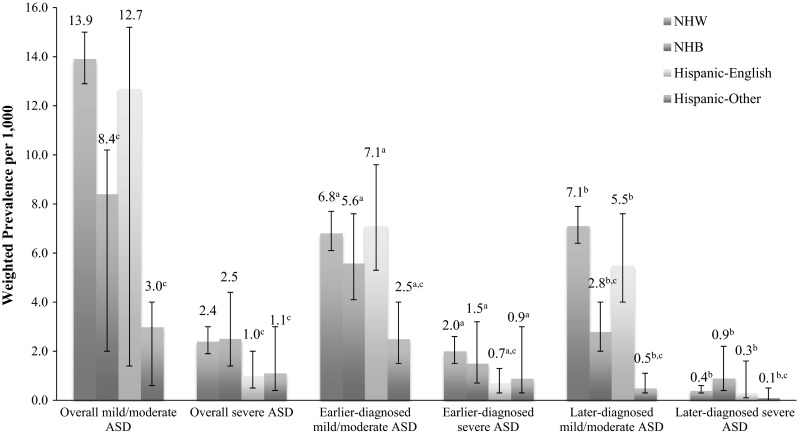
Estimated prevalence of ASD by race/ethnicity/language, parent-assessed severity, and age at diagnosis among children aged 5–17 years at survey, United States, 2009–2010. Weighted prevalence estimates are per 1000 non-institutionalized children in the US population. ^a^Age at diagnosis is <5 years. ^b^Age at diagnosis is ≥5 years. ^c^ *P* < 0.05 two-tailed *z* test comparing the prevalence estimate within each race/ethnicity/language group to the prevalence estimate among NHW children

Given the findings observed in our analyses of diagnosis age, we further assessed ASD prevalence within subgroups based on diagnosis age and severity (Fig. 2). Among children aged 5–17 years, the prevalence differential between NHW and Hispanic-Other children was much larger for mild/moderate ASD (13.9 vs. 3.0) than severe ASD (2.4 vs. 1.1). Likewise, the prevalence differential between NHW and NHB children was notable for mild/moderate ASD (13.9 vs. 8.4), while prevalence of severe ASD was comparable (2.4 vs. 2.5). The prevalence variation across race/ethnicity/language groups was also greater for those with later versus earlier diagnoses and was particularly prominent for those with later diagnoses of mild/moderate ASD.

## Discussion

These findings demonstrate that NHW children have higher reported ASD prevalence estimates and a higher proportion of later diagnoses than NHB and Hispanic-Other children. However, the variation in diagnosis age was limited to children with mild/moderate ASD. Additionally, our detailed assessment of prevalence estimates suggests that while Hispanic-Other children have much lower levels of ASD ascertainment than NHW children at all ages, the prevalence difference is particularly striking for diagnoses of mild/moderate cases at later ages. Further, while the prevalence of earlier-diagnosed ASD is comparable between NHW and NHB children, the prevalence of later-diagnosed mild/moderate ASD is again much higher for NHW than NHB children. Thus, the prevalence disparity between NHWs and two minority groups appears to be primarily driven by under-representation (potentially under-identification) of older children with mild/moderate ASD in the two minority groups.

The pattern of our race/ethnicity-specific prevalence findings are in line with previous studies [7, 9], and our findings for Hispanic children confirm a previous study that found lower ASD prevalence in Hispanic children with foreign-born parents than Hispanic children with US-born parents [26].

However, contrary to some previous studies on racial/ethnic differences in diagnosis age [24, 29], NHB and Hispanic children in our study had a comparable or lower diagnosis age than NHW children. Differences in study results might be explained by methodological differences. While we subdivided Hispanic children based on language, previous studies of diagnosis age treated Hispanic children as a single homogenous group. We also had a much larger sample size and assessed a wider age range than previous studies. We examined later diagnoses in the context of ASD severity while some previous studies did not. However, studies have noted that children with earlier diagnoses were more likely to have prominent clinical characteristics such as language regression [20, 29], which is consistent with our finding of lower diagnosis ages among children reported to have severe ASD. Additionally, a US population-based study reported that children identified with ASD at later ages had fewer ASD characteristics, overall, documented in their school and health records than children identified earlier and children with certain types of ASD features, such as impairments in non-verbal communication, were less likely to have later diagnoses [12].

If the lower prevalence estimates among older NHB and Hispanic-Other children with relatively mild ASD is the result of decreased identification, this might reflect less access to services and limited acculturation among Hispanic-Other children. Previous studies controlling for socioeconomic status suggest that Hispanic immigrants are more likely than non-immigrants to use folk remedies rather than preventive care and mental health services [34, 35]. Hispanic immigrants also encounter cultural and language barriers when seeking health care [35]. Previous research also suggests that NHB and Hispanic children have less access to mental health services than NHW children [36] and that CSHCN living in non-English-primary-language-households are less likely to have a medical home, a usual source of care, and family-centered care than CSHCN from English-primary-language-households [37, 38]. Finally, the 2007 American Academy of Pediatrics recommendations for more aggressive ASD surveillance might have had a larger impact on NHW children with better healthcare access than NHB and Hispanic-Other children [39, 40].

Our finding that children with later ASD diagnoses were more likely than children with earlier diagnoses to have certain co-occurring health conditions was consistent with another recent study [41]. Thus, in addition to access to appropriate diagnostic services, some later diagnoses might be attributable to an early masking effect by the symptoms associated with other conditions.

Strengths of this study include the largest nationally representative sample available to assess this emerging area of research, and availability of data to examine both ASD severity and co-occurring health conditions. We were still, however, limited by small sample sizes within some subgroups. Thus, the presented estimates should be cautiously interpreted. We present these estimates to provide a general sense of the possible absolute variation in ASD diagnosis age across race/ethnicity/language and ASD severity subgroups, and for comparison of ASD prevalence rates with other previously reported rates in US children [9]. Although some point estimates had large RSEs, we note that most of the relative comparisons were fairly stable.

This study also has several other potential limitations. We lacked data on the specific reasons for a later ASD diagnosis. Parent-reported ASD, ASD severity, and co-occurring health conditions were not validated clinically; however, previous studies suggest reliability of parent-reported ASD [7] and comparability between ASD prevalence estimates from national surveys and ADDM [7, 17]. Nonetheless, although our findings are suggestive of under-ascertainment of mild ASD in certain subgroups, we lacked the data to definitely dismiss the possibility that some of the differential between NHW and minority children was due to over-reporting of mild ASD among NHW children. Potential nonresponse bias is possible; however, a previous study demonstrated this likely had little impact on the ASD prevalence estimates [9]. Finally, relative to other US surveys, the NS-CSHCN used a slightly different case definition where parents had to endorse both special healthcare needs and ASD; thus, some children with ASD who were higher-functioning might have been missed. Nonetheless, the ASD prevalence estimates from this study based on 2009–2010 data falls as expected between published estimates from 2007 and 2011 NSCH [9, 11].

## Conclusion

The prevalence of CSHCN with diagnosed ASD remains lower among NHB and non-English-speaking Hispanic children than NHW children; the possibility of under-diagnosis of mild/moderate ASD among older children in these two minority groups needs to be considered. The potential for under-diagnosis is concerning since this will result in delayed treatments. Previous studies suggest early intervention is associated with improved outcomes [3–6]. Although there are less data for children with ASDs who are considered high-functioning, recent reports suggest early elementary school interventions have positive effects on social functioning for this group as well [42]. The findings of this study further support previous recommendations for educating both parents and healthcare professionals about early detection of ASD, and providing culturally competent care [2, 8, 21, 23, 33]. Finally, the results illustrate that studies examining all Hispanic children as a single subgroup might be masking important within-group differences.
